# Bitter Phytochemicals as Novel Candidates for Skin Disease Treatment

**DOI:** 10.3390/cimb46010020

**Published:** 2023-12-30

**Authors:** Teodora-Cristiana Grădinaru, Adelina Vlad, Marilena Gilca

**Affiliations:** 1 Department of Functional Sciences I/Biochemistry, Faculty of Medicine, Carol Davila University of Medicine and Pharmacy, 050474 Bucharest, Romania; teodora.gradinaru@drd.umfcd.ro (T.-C.G.); marilena.gilca@umfcd.ro (M.G.); 2 Department of Functional Sciences I/Physiology, Faculty of Medicine, Carol Davila University of Medicine and Pharmacy, 050474 Bucharest, Romania

**Keywords:** bitter, TAS2R, phytochemicals, skin, skin inflammation, skin cancer, skin aging, wound healing

## Abstract

Skin diseases represent a global healthcare challenge due to their rising incidence and substantial socio-economic burden. While biological, immunological, and targeted therapies have brought a revolution in improving quality of life and survival rates for certain dermatological conditions, there remains a stringent demand for new remedies. Nature has long served as an inspiration for drug development. Recent studies have identified bitter taste receptors (TAS2Rs) in both skin cell lines and human skin. Additionally, bitter natural compounds have shown promising benefits in addressing skin aging, wound healing, inflammatory skin conditions, and even skin cancer. Thus, TAS2Rs may represent a promising target in all these processes. In this review, we summarize evidence supporting the presence of TAS2Rs in the skin and emphasize their potential as drug targets for addressing skin aging, wound healing, inflammatory skin conditions, and skin carcinogenesis. To our knowledge, this is a pioneering work in connecting information on TAS2Rs expression in skin and skin cells with the impact of bitter phytochemicals on various beneficial effects related to skin disorders.

## 1. Introduction

Traditionally viewed as merely a mechanical barrier separating the body from its surroundings, the skin is now recognized as a dynamic, multi-layered interface with diverse functions (e.g., keratogenesis, melanogenesis, hydrolipidic layer formation, sudoral and sebaceous secretion, pilogenesis, thermoregulation, metabolic, endocrine, elastic, plastic, resistance, immunological, psychosocial, and communication functions) [1]. Some of these roles are attributed to the living cells in the skin (e.g., keratinocytes, melanocytes) or to the skin infiltrating cells (e.g., T lymphocytes), while others are achieved in connection with other organs/systems.

Rated as the fourth most prevalent health concern, dermatologic diseases are a substantial healthcare challenge [2]. Inflammatory skin conditions hold a significant weight among them, since approximately one in four individuals will experience a chronic inflammatory skin disease at some point [3]. This category covers various ailments, with psoriasis and atopic dermatitis among the most prevalent [4]. Both conditions profoundly impact the patients’ quality of life and also affect their families [5].

Alongside ultraviolet radiation skin exposure [6], viruses [7], immunodeficiency [8,9], genetic predisposition [10,11], existence of multiple nevi [12], smoking [13], chemical [14] or ionizing radiation exposure [15], chronic cutaneous inflammation is an important risk factor for skin carcinogenesis [16,17].

The incidence of skin cancer has increased in the past few years. In 2020, over 1.5 million new cases of skin cancers (except basal cell carcinoma) have been diagnosed globally [18]. Given the socio-economic impact of inflammatory skin diseases and skin cancers, it becomes imperative to seize every possible opportunity for their prevention and treatment.

A better understanding of the mechanisms by which skin inflammation and cancer are developed may lead to effective therapies. Many of the reported molecules involved in skin inflammation and cancer, such as C–C chemokine receptor type4 (CCR4), C–C chemokine receptor type10 (CCR10), involved in skin lymphocyte recruitment [19], G-protein-coupled estrogen receptor 1 (GPER1) [20], and melanocortin 1 receptor (MC1R), involved in melanocyte proliferation, pigmentary variation, sun sensitivity, and susceptibility to skin cancers [21,22], are G-coupled protein receptors (GPCRs). Based on this, scientists have directed their interest toward other proteins within the same class, identifying them as potential targets for drug development in treating skin diseases. Among them, taste receptors (TASRs), especially bitter taste receptors (TAS2Rs), discovered relatively recently to have widespread extraoral expression (including in the skin), are a novel class of GPCRs that became attractive for scientists in dermatology.

Exploring natural resources through bioprospecting, an important research trend in dermatology [23,24,25], may be a useful approach for identifying new ligands of TASRs that could serve as potential templates for the development of innovative drugs and therapeutic strategies for various skin diseases. Many natural compounds exert anti-inflammatory effects, acting on specific molecular pathways involved in the development of skin inflammation and/or its progression to cancer [26,27]. Among them, bitter phytochemicals showed a greater probability of possessing both anti-inflammatory and anticancer properties than other plant-derived tastants [28,29].

Recent research has highlighted the involvement of TAS2Rs in inflammatory conditions [30,31,32] and their differential expressions in various tumors [33,34], suggesting potential as therapeutic targets for these diseases. Additionally, increasing evidence indicates the therapeutic potential of bitter compounds in anti-cancer strategies [33,35]. While TAS2Rs have been identified in skin samples and cells, their specific metabolic pathways and functions remain to be clearly defined.

This review aims to primarily summarize evidence concerning the following aspects: (1) the expression of TAS2Rs in the skin; (2) the physiological and pathophysiological roles of TAS2Rs in human skin; and (3) the influence of the most representative bitter phytochemicals, known as TAS2R agonists, on various skin processes such as keratinocyte differentiation, skin aging, wound healing, inflammation, and skin carcinogenesis. We also aim to assess the extent to which these biological activities of bitter phytochemicals may be mediated by TAS2Rs and to propose the hypothesis that targeting inflammation via the TAS2Rs signaling pathway could potentially prevent skin carcinogenesis.

## 2. Bitter Taste Receptors (TAS2R)-Types and Signaling Pathways

Bitter taste represents one of the five basic tastes (bitter, sweet, sour, salty, umami). From an evolutionary point of view, bitter taste is thought to play an important role in avoiding potentially harmful substances, which most frequently taste bitter [36,37].

To date, 26 human TAS2Rs are known [38]. These receptors belong to the type A G protein-coupled receptor cluster [39]. The binding of bitter tastants to TAS2R induces a conformational modification of this receptor, followed by the dissociation of G-protein in Gα-gustducin and Gβγ [40,41]. Gβγ activates the β2 isoform of phospholipase, capable of converting phosphatidylinositol 4,5-bisphosphate (PIP2) into inositol 1,4,5-trisphosphate (IP3) and diacylglycerol (DAG) [40,41]. Upon binding to its receptor located on the surface of the endoplasmic reticulum (ER), IP3 triggers calcium efflux from the ER, leading to an increase in the cytosolic concentration of Ca^2+^ [40,41]. Subsequent molecular events depend on the specific cell type. In the taste buds, a rise in cytosolic Ca^2+^ concentration determines membrane depolarization through transient receptor potential cation channel subfamily M member 4/5 (TRPM4/5), prompts ATP release from calcium homeostasis modulator 1/3 (CALHM1/3), and initiates the activation of nerve fibers that transmit the gustatory signal to the brain [40,41].

In the skin, the TAS2R signaling pathway is not yet completely elucidated. Activation of this receptor in skin cells leads to an intracellular increase in Ca^2+^ concentration [42].

Among the 26 known human bitter taste receptors, four are considered orphan receptors (TAS2R42, TAS2R45, TAS2R48, and TAS2R60), indicating that their specific activating compounds or ligands have not yet been identified [43,44,45,46,47,48]. Some of the remaining 22 exhibit broad tuning, displaying varying levels of promiscuity, such as TAS2R10 [43], TAS2R14 [43,49], TAS2R39 [43], and TAS2R46 [43,50]. Others demonstrate narrow modulation, responding to a limited set of agonists; for example, TAS2R3 recognizes chloroquine and theaflavin-3′-O-gallate [43,51], while TAS2R41 acts as a ‘specialist’ receptor for chloramphenicol [46]. In contrast, certain compounds stimulate multiple TAS2Rs—for example, quinine activates 9 TAS2Rs [43], and yohimbine stimulates 10 TAS2Rs [38,43]—while other substances selectively target only one TAS2R as specific agonists.

To date, there are several freely available online databases containing information about bitter-tasting compounds and their affinity for various TAS2Rs. Bitter DB (http://bitterdb.agri.huji.ac.il, accessed on 1 October 2023), initially published in 2012 and updated in 2018, is dedicated to all types of bitterants (synthetic and natural), containing more than 1000 bitter molecules. Data on the associated TAS2Rs are available for 270 compounds [52]. PlantMolecularTasteDB (https://plantmoleculartastedb.org/, accessed on 1 October 2023) is dedicated to all types of plant-derived orosensorially active compounds (bitter, sweet, sour, salty, umami, pungent, and astringent) and contains more than 1000 bitter phytocompounds, among which 180 have documented ligand properties for one or more TAS2Rs [53]. Also, online tools for bitter taste prediction and TAS2R activation have been developed [54,55].

## 3. Extraoral Bitter Taste Receptors and Their Biological Roles

TAS2Rs are expressed in virtually all human systems, including cardiovascular [56,57], digestive [58,59,60], endocrine [61], immune [62,63], integumentary [42,64,65,66], muscular [67], nervous [68,69,70,71], renal [72,73], reproductive [74,75,76], respiratory [77,78,79], and skeletal systems [80,81,82,83]. Evidence shows an increasing number of cells expressing TAS2Rs. Interestingly, recent studies have highlighted differential expression of TAS2Rs in inflammatory versus non-inflammatory states [84,85], as well as in normal versus cancerous cells [86,87].

The discovery of extraoral taste receptors has raised questions regarding their biological roles in these cvasi-ectopic locations. Beyond their primordial attribute of detecting bitter taste, TAS2Rs have various non-sensorial functions. They are involved in regulating innate and adaptive immunity [63,88,89,90,91], inflammation [31,92], endocrine or exocrine secretion [61,93,94], or the contraction/relaxation process [95,96]. As well, they are engaged in the pathogenesis of various diseases, such as cancer [33,34,76,97], metabolic disorders [98,99,100], and infections [101,102].

Furthermore, a limited number of studies have demonstrated that TAS2Rs can bind to endogenous ligands, including bile acids [103] or progesterone [47], suggesting additional physiological roles that are yet to be revealed.

## 4. Differential Expression of Bitter Taste Receptors in Human Skin

The expression of TAS2Rs in human skin varies significantly based on diverse factors such as the degree of sun exposure, physiological characteristics (e.g., sex, age), or the presence of pathological conditions (e.g., inflammation) in the tested specimens, as outlined in Table 1. For instance, in sun-exposed skin samples (lower leg) compared to non-exposed areas (suprapubic), *TAS2R14*, *TAS2R30*, and *TAS2R42* are notably less expressed, while *TAS2R60* exhibits a significant increased expression [64]. Moreover, a positive correlation occurs in non-exposed skin areas between aging and *TAS2R5* expression levels [64]. Women display significantly higher expression levels of *TAS2R3*, *TAS2R4*, and *TAS2R8* in the suprapubic area (considered non-exposed skin), while exhibiting lower levels of *TAS2R3*, *TAS2R9*, and *TAS2R14* in sun-exposed regions like the lower leg; *TAS2R60*, however, has a significantly higher expression [64].

Scientists also reported high inter-individual variability in *TAS2Rs expression*, with some having universal expression across individuals, although at varying levels, while others were expressed selectively [64,65]. In addition, *TAS2Rs* show differential expression based on the investigated sample types, including whole skin samples, cell lines, or primary cells. For instance, the *TAS2R14* transcript had a notably higher expression in human skin samples compared to primary basal keratinocytes and both differentiated and undifferentiated N/TERT-1 keratinocytes [42]. Immunofluorescent analysis using anti-TAS2R14 antibodies displayed the strongest signal in the stratum granulosum of skin samples, whereas N/TERT-1 keratinocytes presented a signal dispersed throughout the cytoplasm [42].

The expression of bitter taste receptors covers various skin cellular types, supported by positive evidence in keratinocytes [42,66,73], fibroblasts [73], adipocytes [106], and other resident inflammatory cells strategically located in the skin, such as infiltrating lymphocytes [107] (Table 1). Therefore, it can be concluded that *TAS2Rs* are expressed across all strata of the skin: epidermis, dermis, and hypodermis.

These observations highlight a nuanced and personalized profile of bitter taste receptor expression across different skin conditions and demographics.

## 5. Functionality of Bitter Taste Receptors in Skin

There is substantial evidence regarding the functionality of TAS2Rs expressed in the skin. Their activation by corresponding agonists (e.g., (-)-α-thujone for TAS2R14 [42], amarogentin for TAS2R1 [66]) is associated with a dose-dependent increase in intracellular calcium concentration. Several studies have indicated potential ligands for skin TAS2Rs, suggesting that besides exogenous compounds (both natural and pathogenic) [108], endogenous substances could also activate these receptors, including a keratinocyte-derived product not yet identified [107] or bitter amino acids derived from natural moisturizing factors [108].

Skin TAS2Rs are believed to display diverse biological roles as chemosensory receptors, regulators of keratinocyte differentiation, skin barrier protein expression and lipid synthesis, inhibitors of hair growth, modulators of skin aging, wound healing, adipocyte functions, migration within the skin, and oral microbiota [42,104,105,109,110].

Upon reviewing the existing literature on TAS2Rs, we discovered a lack of comprehensive studies regarding their general functionality, particularly in the skin. Furthermore, there is no consensus on the role of certain TAS2Rs, such as TAS2R5 (see reference [111]). In response to this gap, we have synthesized the available evidence on their established roles in both the skin and oral cavity, as presented in Table 2.

### 5.1. Bitter Taste Receptors as Chemosensory Receptors

Alongside Merkel cells, keratinocytes have the ability to receive external sensory stimuli and trigger skin sensations, including nociception [117,118]. There is a suggestion among scientists that TAS2Rs might act as chemosensory receptors in skin cells, allowing them to recognize noxious compounds that may have breached a damaged epidermal barrier. For instance, TAS2R14, expressed in keratinocytes, recognizes (-)-α-thujone, a well-known neurotoxin, and may confer on the cells the ability to identify hazardous chemicals [42]. Using a knockout keratinocyte cell model, Kung-Yu Ho et al. demonstrated that α-thujone-induced Ca^2+^ signals rely on wild-type *TAS2R14*, and pharmacological inhibition by suramin points to the involvement of heterotrimeric G proteins in the signaling pathway [42].

### 5.2. Bitter Taste Receptors as Regulators of Keratinocyte Differentiation and Skin Barrier Structural and Functional Integrity

Certain bitter compounds, such as amarogentin (a non-selective TAS2R1 agonist) and diphenidol (an agonist for TAS2R1 and TAS2R38), have been found to induce the expression of both early and late differentiation markers in human primary keratinocytes and HaCaT cells (markers including Keratin 10, involucrin, and transglutaminase-1) [66]. These compounds not only influence skin barrier proteins but also impact skin lipids. For example, in a separate study, *Gentiana lutea* extract increased lipid synthesis in keratinocytes by activating the peroxisome proliferator–activated receptor γ (PPAR-γ) and p38 mitogen-activated protein kinase (p38 MAPK) pathways and induced the expression of ceramide synthase 3 (CerS3), a process also dependent on PPAR-γ and p38 MAPK [109]. A 5% *Gentiana lutea* extract cream applied twice daily for 4 weeks to the volar forearms of 33 healthy volunteers determined an increased lipid content in 28 of the participants [109]. Furthermore, in the same study, *Gentiana lutea* extract did not trigger the release of pro-inflammatory mediators such as PGE2 and IL-6 from human primary keratinocytes [109]. Regarding ceramide metabolism, studies reported that some of the human primary keratinocytes were non-responsive to stimulation with the bitter extract of *Gentiana lutea*, a reaction that may be due to the occurrence of polymorphisms in bitter taste receptors [66,109].

### 5.3. Influence of Bitter Taste Receptors on Aging and Wound Healing

The aging process in the skin can be induced by D-galactose through various molecular mechanisms involving oxidative stress: downregulation of antioxidant enzymes; formation of advanced glycation end products (AGEs) that target extracellular matrix proteins, such as collagen and elastin, diminish their quality and quantity and subsequently cause reduced skin flexibility; activation of NADPH oxidase; and increase of mitochondrial DNA damage, among others [119]. Intriguingly, in D-galactose-induced aged HaCaT keratinocytes, *TAS2R10* and *TAS2R16*, along with downstream proteins (TRPM5 and PLCβ2), had increased expression compared to normal HaCaT cells [105]. Similarly, in a D-galactose-induced aged mouse model, the mouse counterparts of human *TAS2R16 (Tas2r118)* and *TAS2R10 (Tas2R114)* were found to be overexpressed in the skin relative to normal mouse skin [105]. In D-galactose-treated HaCaT cells transfected with *TAS2R16*, its overexpression reduced the relative levels of skin aging markers (p53, p21) and increased the relative expression of antioxidant enzymes (superoxide dismutase 1, glutathione peroxidase 1, and catalase) when compared to D-galactose-treated HaCaT cells [105].

Scientists have also evaluated the impact of overexpressed TAS2R16 on wound healing by using a scratch wound-healing assay based on D-galactose-transfected *TAS2R16* HaCaT cells [105]. TAS2R16 overexpression reduced the wound width, increased the relative expression of various collagenolytic enzymes (MMP-2, MMP-9), and decreased the relative expression of tissue inhibitor of metalloproteinases 2 (TIMP-2). TAS2R16 overexpression also induced markers of the epithelial mesenchymal transition process, essential for wound repair, by decreasing E-cadherin expression and increasing mesenchymal markers (N-cadherin, vimentin) [105,120]. D-galactose-treated HaCaT cells transfected with *TAS2R10* displayed narrower wound widths compared to D-galactose-treated HaCaT cells, indicating an enhanced capacity for wound healing [105]

### 5.4. Bitter Taste Receptors as Regulators of Hair Follicle Growth

The hair follicle growth cycle comprises three phases: 1. anagen, characterized by active cell division and hair growth, lasting 3–10 years; 2. catagen, marked by the cessation of cell division and hair growth, completed in 2–3 weeks; 3. telogen, the resting phase, with a duration of 3–4 months [121]. Keratinocytes from the outer root sheath of scalp hair follicles express functional TAS2R4.. Stimulation of TAS2R4 with rebaudioside A has been found to prematurely induce the catagen phase through TGF-β2, thus inhibiting hair growth [104].

### 5.5. Bitter Taste Receptors as Modulators of Skin Immunity and Oral Microbiome

Bitter taste receptors play a role in skin immunity. Functional TAS2R38 receptors are expressed by skin-infiltrating lymphocytes [107]. Both mRNA and protein expression levels were significantly higher in atopic dermatitis compared to healthy skin [107]. This receptor can be considered a marker for the severity of atopic dermatitis based on the positive correlations established between serum thymus and activation-regulated chemokine (TARC), or serum IgE and *TAS2R38* mRNA levels [107]. Stimulation of TAS2R38 with phenylthiocarbamide (PTC) and 6-n-propylthiouracil (PROP) induces a dose-dependent inhibition of the migration signal in *TAS2R38*-transduced Jurkat cells in response to CXCL12 [107]. Interestingly, HaCaT cell culture supernatants and skin extracts have been found to contain an endogenous TAS2R38 ligand that inhibits migration [107].

The *TAS2R9* gene and TAS2R9 protein were identified through a DNA microarray and antibody array conducted on the HaCaT cell line, which overexpressed aldehyde dehydrogenase 1 (ALDH1), as one of the genes, respectively hub proteins, associated with the function of ALDH1 in keratinocytes [113]. Interestingly, transcriptional as well as translational downregulation of ALDH1 was related to atopic dermatitis [122,123], and was proposed as a new potential marker for this disease [113].

Significant associations among several genetic variants in *TAS2Rs* (*TAS2R3*, *TAS2R38*, and *TAS2R41*) and the relative abundances of bacterial or fungal taxa were identified (Table 2) [110].

### 5.6. Bitter Taste Receptors as Regulators of Adipocyte Functions

Chronic exposure of subcutaneous adipocytes to the bitter compound propylthiouracil increased *TAS2R38* expression while concurrently decreasing the expression of three genes involved in adipocyte differentiation (*FASN, GLUT4*, and PPAR-*γ*) [106]. In a separate study, two other bitter agonists, denatonium benzoate and quinine, hampered the differentiation of 3T3-F442A pre-adipocytes into mature adipocytes [124]. This inhibition resulted in reduced expression levels of several differentiation markers, such as leptin, adiponectin, PPAR-γ, adipocyte protein 2, fatty acid synthase, and uncoupling protein 2 [124]. Furthermore, acute stimulation with the bitter compounds caffeine, propylthiouracil, and quinine in differentiated subcutaneous adipocytes led to a decrease in lipid content [106]. Considering the ability of bitter agonists to reduce adiposity in obese mice, researchers have suggested the potential of TAS2Rs as druggable targets to induce body weight loss [124].

### 5.7. Involvement of Bitter Taste Receptors in Skin and Oral Cancers

Increasing evidence suggests a potential link between aberrant expression or mutations of *TAS2Rs* and skin as well as oral cancers.

#### 5.7.1. Skin Melanoma

In a study conducted by Ryan Carey et al., it was found that among all types of cancer studied, skin melanoma exhibited the highest rate of non-silent mutations for *TAS1R* and *TAS2R* [87]. Among TAS2R types, the most frequent non-silent mutations involved *TAS2R38*, *TAS2R41*, and *TAS2R60* [87]. This study also investigated *TAS1R* and *TAS2R* gene expression and their impact on survival rates [87]. Interestingly, increased expression of *TAS2R14* was associated with notably longer survival (over 7 years) in distantly metastatic skin melanoma cases [87]. Even though melanoma originates from melanocytes [125], current genome-wide analysis of tissue-specific RNA and protein expression does not provide evidence of *TAS2R* expression in melanocytic cells (https://www.proteinatlas.org, accessed on 1, October 2023) [126].

#### 5.7.2. Oral Squamous Cell Carcinoma

Oral squamous cell carcinoma, much like skin squamous cell carcinoma, originates from keratinocytes but exhibits certain differences in evolution and management [127,128]. In some samples from oral cavity squamous cell carcinoma and corresponding contralateral normal locations, intraindividual variations in *TAS2R* expression were observed [35]. Differential expressions for *TAS2Rs* were also observed in oral squamous cell carcinoma cell lines [35]. Interestingly, TAS2Rs are expressed on the nuclear membrane, and upon activation, they induce an increasing calcium nuclear concentration, leading to mitochondrial dysfunction and apoptosis [35]. In a study analyzing the Cancer Genome Atlas for head and neck squamous cell carcinoma, Carey et al. found a positive correlation between high *TAS2R* expression and overall survival and *TAS2R4* expression and overall survival rates, respectively [35].

### 5.8. Other Potential Functions of Bitter Taste Receptors

Indirect evidence suggests various potential functions of TAS2Rs. For example, recent research demonstrated the involvement of several TAS2Rs (TAS2R3, TAS2R4, TAS2R14, TAS2R19, and TAS2R43) expressed in follicular granulosa cells in gonadal steroidogenesis [129]. Considering the established elevated expression of *TAS2R3* and *TAS2R4* in the female suprapubic area, an area abundant in sexual hair follicles [64], and the recognition of skin and hair follicles as sites for extra-adrenal and extra-gonadal steroidogenesis [130], we can estimate a potential role for TAS2R3 and TAS2R4 in cutaneous steroidogenesis and hair follicle growth. Notably, the regulatory role of TAS2R4 in hair follicle growth has already been experimentally confirmed [104].

## 6. Chemical and Orosensorial Complexity of Bitter Phytochemicals

Bitter phytochemicals are characterized by significant structural heterogeneity, belonging to various chemical classes: alkaloids, aminoacids, carotenoids, coumarins, flavonoids, steroids, terpenoids, etc. [53]. The majority of bitter compounds are characterized by their relatively small molecular size and high hydrophobicity, notably distinct from sweet compounds, which are generally larger and more polar [54].

A single phytochemical may present a complex orosensory profile, inducing multiple tastes or orosensations. Compounds that display simultaneous bitter taste and astringency are often found in the tannin class (e.g., castalagin activates TAS2R7 [131]) or within flavonoids (e.g., myricetin activates TAS2R14 and TAS2R39 [132]). Camphor, a well-known pungent monoterpenoid, also activates several bitter taste receptors: TAS2R4, TAS2R10, TAS2R14, and TAS2R47 [43]. Certain sulfur compounds, despite their pungency, act as ligands for various TAS2Rs as well (e.g., allyl isothiocyanate activates TAS2R38, sinigrin activates TAS2R16 [43]).

Although the majority of saccharides are sweet, a few display bitterness (e.g., gentiobiose [133], gentianose [134], beta-D-mannose) [135]). Rebaudiosides, belonging to the class of steviol glycosides, are well known as sweet compounds, but many of them also exhibit slight bitterness, acting as agonists for various bitter taste receptors (e.g., rebaudiosides A, B, and C are agonists of TAS2R4 and TAS2R14 [136]).

## 7. Bitter Phytochemicals Active on Skin Inflammation, Skin Carcinogenesis, and Wound Healing

Recently, we have demonstrated that taste serves as a more significant predictor of anti-inflammatory and anti-cancer activity than the chemical class itself [28,29,137]. Bitter taste was positively correlated, while sweet taste showed a negative correlation with both activities [29]. As outlined in a recent review, anti-inflammatory activity emerged as the most frequently cited biological property of natural compounds beneficial in wound healing [138]. This finding is reasonable considering that inflammation represents the second phase of the wound healing process [138]. Therefore, bitter phytochemicals show promising therapeutic potential in treating skin inflammatory diseases, skin cancer, and skin ulcers.

**Figure 1:**
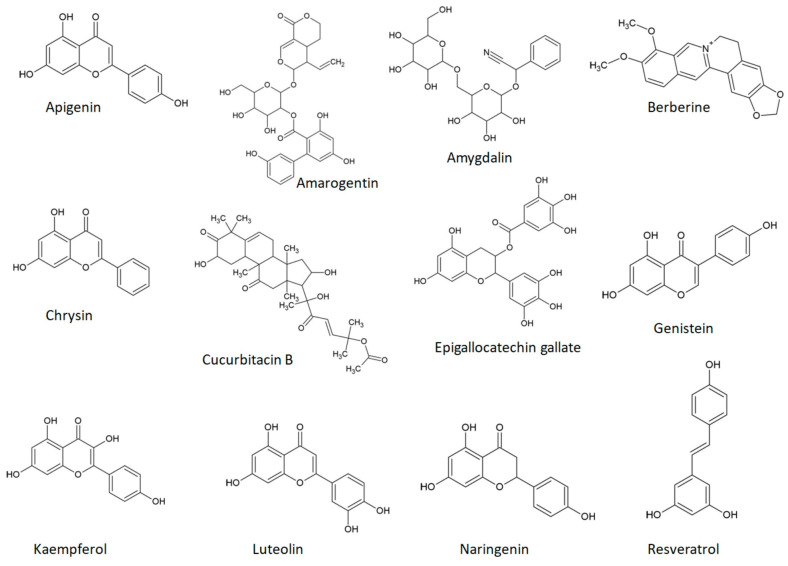
Chemical structures of some bitter phytochemicals.

Figure 1 illustrates the chemical structures of a select group of bitter phytochemicals, which will be briefly discussed in connection with TAS2Rs in this section.

Apigenin, also known as 4′,5,7-trihydroxyflavone, is a bitter flavonoid present in fruits, vegetables, seasonings, medicinal plants, or plant-derived beverages (e.g., grapefruit, lettuce, celery, parsley, oregano, rosemary, red and white wine [139,140,141]). Apigenin is a well-known agonist of TAS2R14 [132,142] and TAS2R39 [132]. This flavonoid has been associated with various pharmacological activities, such as anti-oxidant [143], anti-inflammatory [144,145,146], anti-cancer [147,148], anti-bacterial [149], anti-viral [150,151], cardioprotective [152], and anti-obesity effects [153].

In the skin, apigenin showed beneficial effects in addressing inflammatory conditions and certain types of skin cancer. In a mouse model of imiquimod-induced psoriasis-like skin lesions, apigenin decreased erythema, scaling, and Psoriasis Area and Severity Index (PASI) score, and it also inhibited NF-kB activation and the IL-23/STAT3/IL-17A pathway [154]. Furthermore, apigenin decreased protein expressions of TNF-α, IL-1β, and IL-6 at the skin level [154]. Similarly, in a mouse model of induced atopic dermatitis, apigenin decreased skin lesions, alleviated cutaneous symptoms, and reduced IgG1 and IgE levels in mouse serum [155]. In studies involving skin cancer cell lines, apigenin had inhibitory effects on growth, proliferation, survival, invasion, and migration while inducing apoptosis and cytotoxicity [156,157,158,159,160,161,162]. Furthermore, in mouse models of skin cancer, apigenin decreased tumor growth [158,159]. In a mouse skin carcinogenesis model, apigenin inhibited tumor development and delayed tumor appearance [163].

Amarogentin, identified as an agonist of seven TAS2Rs (TAS2R1 [43], TAS2R4 [43], TAS2R39 [43], TAS2R43 [43], TAS2R46 [43], TAS2R47 [43], TAS2R50 [43]), is a secoiridoid glycoside that can be found in various plants from the Gentianaceae family and ranks among the most bitter natural substances [164,165,166]. In a mouse model of induced atopic dermatitis, amarogentin decreased IgE serum levels and demonstrated anti-inflammatory effects [167]. In another mouse model of skin carcinogenesis, the amarogentin-rich fraction from *Swertia chirata* exerted proapoptotic and antiproliferative actions [168]. Additionally, amarogentin was observed to inhibit substance P-mediator release of TNF-α and block the secretion of newly synthesized TNF-α from LAD-2 mast cells [108]. Scientists suggested that these effects might be mediated by TAS2R1, which has been confirmed to be expressed by mast cells. In HaCaT cells, amarogentin indirectly inhibited MMP-1 and IL-8 secretion through TNF-α and histamine pathways [108].

Amygdalin, also known as D-mandelonitrile-β-D-gentiobioside or vitamin B17, is a cyanogenic glucoside notably present in the seeds of various species within the Rosaceae family (e.g., apricot, peach, bitter almond) [169] that acts as an agonist of TAS2R16 [43,170]. Amygdalin proved to have good skin penetration, making it a good candidate for skin diseases [171]. In a study using burn-induced skin wounds in diabetic rats, amygdalin improved the time and quality of wound healing [172]. However, due to the in vivo release of hydrogen cyanide, amygdalin possesses a certain degree of toxicity (LD50 of approximately 522 mg/kg in rats) [173]. As a result, less toxic analogues of amygdalin have been developed. These amygdalin analogues showed in vitro several immunoregulatory effects in human epidermal keratinocytes that may exert potential healing actions in psoriasis (e.g., upregulation of IL-10, HSP-70, TGF-beta, alpha-v integrin, inhibition of IFN-γ signaling, downregulation of ICAM-1 expression) [174,175].

Systemic administration of these amygdalin analogues in a xenograft transplantation model, where human psoriatic skin was transplanted onto immunodeficient mice, significantly improved psoriatic lesions [169]. This improvement was reflected in the reduction of clinical psoriasis score, epidermal thickness, parakeratosis, and Munro’s abscesses [169]. Also, topical application of a cream based on an amygdalin analogue ameliorated psoriasis-like disease in a mouse model [176]. It achieved this by reducing keratinocyte proliferation, skin inflammation, and the levels of systemic pro-inflammatory cytokines typically increased in psoriasis subjects, such as IL-17A, IL6, or G-CSF [176].

Berberine, a bitter alkaloid found in various plants, acts as an agonist for TAS2R38 [177] and TAS2R46 [178]. In an animal model of atopic dermatitis, berberine displayed anti-inflammatory effects [179]. In the context of skin cancer, a growing body of evidence supports the antitumor actions of berberine. In both melanoma and squamous cell carcinoma cells, berberine inhibited cell proliferation, migration, and invasion, inhibited epithelial-mesenchymal transition, and induced apoptosis [180,181,182]. In an in vivo model of well-differentiated squamous cell carcinoma, berberine increased the activity of antioxidant enzymes such as superoxide dismutase (SOD) and glutathione peroxidase (GPx) [183].

Chrysin, a flavonoid also known as 5,7-dihydroxyflavone, is a TAS2R14 [132] and TAS2R39 [132] agonist. It can be found in certain medicinal plants, such as *Opuntia ficus indica* L and *Viburnum opulus* L [184,185], as well as in honey [186] and propolis [187]. In an imiquimod-induced mouse psoriasis model, chrysin demonstrated an ability to alleviate inflammation [188]. In another mouse model of atopic dermatitis, chrysin reduced serum histamine and IgE levels, inhibited the inflammatory response, and decreased mast cell infiltration [189]. Regarding skin cancer cells, chrysin inhibited cell proliferation, migration, invasion, metastasis, induced apoptosis, and reduced angiogenesis and mTOR expression while increasing caspase-3 activity [190,191,192,193,194,195,196,197]. In a mouse model of skin carcinogenesis, chrysin decreased tumor formation, volume, and number and had a stimulatory effect on the activity of certain antioxidant enzymes (e.g., SOD, GPx) [198]. In a mouse skin cancer model, chrysin inhibited tumor growth and decreased tumor size and volume [193]. Also, in an animal model of skin cancer, chrysin reduced the metastatic potential [195].

Cucurbitacin B, a triterpenoid known to act as an agonist for TAS2R10 [43] and TAS2R14 [43], shows promising anti-inflammatory and anti-cancer actions specific to the skin. In a model of imiquimod-induced skin inflammation, cucurbitacin B inhibited psoriatic cytokines (e.g., IL-8, CCL-20) and keratinocyte proliferation [199]. In both in vitro experiments (using squamous carcinoma cells or human or murine melanoma cells) and in vivo studies (employing murine models), cucurbitacin B exhibited potent anti-cancer effects [200,201,202,203]. It induced cell cycle arrest in the G2/M phase, inhibited cancer cell proliferation and migration, demonstrated cytotoxic activity, and reduced tumoral volume and growth [200,201,202,203].

Epigallocatechin gallate (EGCG), a flavonoid and known agonist for TAS2R14 [132,204], TAS2R31 [204], and TAS2R39 [132,204], is the major component in green tea [205]. It demonstrates beneficial effects concerning skin inflammation and cutaneous tumors. In a mouse model induced by psoriasis-like inflammation, EGCG reduced the serum level of pro-inflammatory cytokines and mitigated dermal T cell infiltration [206]. Additionally, in a mouse model of induced atopic dermatitis, EGCG lowered IgE serum levels and decreased mRNA expression of TNF-α, MIF (macrophage migration inhibitory factor), IFN-γ, IL-2, and IL-12 in skin lesions [207]. Within skin cancer cells, EGCG induced apoptosis and cell cycle arrest, inhibited growth and proliferation, and reduced clonogenic capacity [208,209,210,211]. In terms of skin carcinogenesis, EGCG reduced tumor number and multiplicity while increasing antioxidant enzymes like SOD and glutathione peroxidase [212]. In mouse skin cancer models, EGCG decreased tumor weight and growth and inhibited metastasis [209,213,214,215]. Furthermore, EGCG displayed beneficial effects on wound healing [216].

Genistein, a soy isoflavone [217] and a recognized agonist for TAS2R14 [132] and TAS2R39 [132], has proven anticancer effects on skin cancer cell lines. It inhibited growth, proliferation, and migration, reduced cell survival and invasion, induced apoptosis, and led to cell cycle arrest [218,219,220,221,222,223,224]. In a mouse skin cancer model, genistein reduced tumor volume [218]. In a two-stage mouse skin carcinogenesis model, it decreased tumor incidence and multiplicity [225]. In an animal model, genistein promoted wound healing [226]. In an experimental model of psoriasis, genistein suppressed Th1 and Th17 cytokines and ameliorated mouse skin lesions [227]. This compound also showed beneficial effects in a mouse model of atopic dermatitis [228].

Kaempferol, a known agonist of TAS2R14 [132] and TAS2R39 [132], is found abundantly in green leafy vegetables [229]. In a mouse model of imiquimod-induced psoriasis, kaempferol reduced T cell infiltration in the skin and the gene expression of inflammatory cytokines [230]. Likewise, in a mouse model of induced atopic dermatitis, kaempferol inhibited inflammatory cell infiltration, reduced inflammation, and decreased involucrin expression [231]. In skin cancer cells, kaempferol inhibited proliferation, migration, metastasis, induced apoptosis, and caused cycle cell arrest [232,233,234]. In a mouse melanoma model, kaempferol reduced both tumor volume and weight [235].

Luteolin is a recognized agonist of TAS2R14 [132] and TAS2R39 [132]. In a murine model of atopic dermatitis, luteolin reduced inflammation, oxidative stress, and serum IgE levels [236]. Similarly, in a mouse psoriasis model, luteolin inhibited the infiltration of inflammatory cells in the skin, decreased levels of proinflammatory cytokines, and lowered inflammatory mediators [237]. Luteolin has shown inhibitory effects on cancer cell lines by reducing proliferation, migration, and invasion, by inducing apoptosis and cell cycle arrest, and by decreasing cell viability [238,239,240,241,242,243,244]. In mouse models of skin carcinogenesis, luteolin inhibited tumor incidence, decreased tumor multiplicity, and reduced tumor volume [245,246]. In mouse skin tumors, luteolin inhibited tumor growth, decreased tumor volume and weight, and suppressed the expression of MMP-2 and MMP-9 [241,242,243].

Naringenin, also known as 4′,5,7-trihydroxyflavone, is a citrus flavonoid identified as a TAS2R14 agonist [52,247]. In a mouse model of induced atopic dermatitis, naringenin inhibited T cell production of IFN-γ, immune cell infiltration into skin lesions, and decreased serum IgE concentration [248]. In skin cancer cell lines (both human and murine), naringenin reduced cell viability and migration and induced apoptosis [249]. In a two-stage mouse skin carcinogenesis model, naringenin decreased the number and size of tumors [250]. In addition, naringenin lowered the number of lung metastases and delayed the mortality of mice inoculated with B16-F10 cells [251]. In the same study, naringenin exhibited a decrease in melanoma cell growth [251].

Resveratrol, a phytoalexin known for its activation of TAS2R14 [132] and TAS2R39 [132], can be found in grapes, wines, and peanuts [252]. It exerts various pharmacological actions, including effects at the cutaneous level. Resveratrol significantly diminished skin inflammation in a mouse model of induced psoriasis [253], and numerous studies highlight its anti-inflammatory roles in models of atopic dermatitis [254,255].

Several studies emphasize the anticancer actions of resveratrol at the cutaneous level. In skin cancer cells (melanoma and squamous cell carcinoma cell lines), resveratrol inhibited cell viability, growth, proliferation, migration, invasion, induced apoptosis, and caused cell cycle arrest [256,257,258,259,260,261,262,263,264,265,266,267]. In mouse models of skin carcinogenesis, resveratrol decreased tumor incidence, among others [268,269]. In mouse models of cutaneous cancers, resveratrol inhibited tumor growth, lowered tumor volume and weight, suppressed metastasis tendency, and increased survival time [257,258,261,265,270,271]. Also, resveratrol displayed beneficial effects in wound healing [272].

Concerning the general disadvantages of phytochemicals, uncertainties arise from insufficient knowledge about the specific targeted pathways, pharmacokinetics, drawbacks, and human pharmacodynamic activities. Additionally, certain phytochemical classes, such as phytoestrogens, pose specific shortcomings, particularly concerning their impact on the reproductive system.

It Is essential to recognize that “natural” does not equate to “safe.” A case in point is amygdalin, which is toxic due to the generation of hydrogen cyanide [173].

Various limitations may emerge based on the route of administration. For example, degradation in the digestive tract or liver biotransformation of phytochemicals during oral administration can negatively affect their bioavailability. In the context of topical administration for skin conditions, different challenges arise; some bitter phytochemicals exhibit limited skin penetration and bioavailability due to low solubility in excipients, while others may induce skin irritation.

Table 3 succinctly outlines the potential indications and drawbacks of these bitter phytochemicals.

## 8. Phytochemical Bitter Taste Receptors agonists in Skin Aging, Inflammation, and Cancer: Insights into mammalian Target of rapamycin (mTOR) Signaling Pathways

Skin aging is a risk factor for skin carcinogenesis, attributed not only to photoaging or environmental exposure but also to intrinsic aging processes [292]. With age, proteins like keratin 10 and involucrin exhibit reduced expression in the epidermis, among other changes [292]. The mTOR pathway plays a pivotal role in aging, including skin aging [293]. This pathway is implicated in both skin inflammatory diseases (e.g., psoriasis) and skin cancers (e.g., melanoma, cutaneous T cell lymphomas) [294].

Numerous bitter phytochemicals exhibit the ability to inhibit the mTOR signaling pathway, thereby influencing skin aging, inflammation, and cancer. For instance, fisetin, an agonist of TAS2R [132], demonstrates anti-inflammatory, anti-proliferative, and pro-differentiation effects in keratinocytes by modulating the mTOR pathway [295]. Resveratrol, by inhibiting the PI3K/AKT/mTOR pathway in melanoma cells (human-A375 cells, mouse-B16-F10 cells), promotes autophagy [260]. Additionally, UVB radiation, a leading factor in skin carcinogenesis, triggers the mTOR signaling pathway, which can be attenuated by the bitter compound apigenin [296]. Berberine, by inhibiting the induced expression of MMP-9 and IL-6 in normal human keratinocytes, exhibits potential as an anti-aging agent for the skin [297].

The specific role of TAS2R-mediated pathways in the development of these interconnected skin conditions—aging, inflammation, and tumorigenesis—remains, though, to be investigated. Such endeavors may open new therapeutic perspectives for the use of TAS2R natural or synthetic agonists in curing skin ailments.

## 9. Direct Involvement of Bitter Taste Receptors in the Bitter Phytochemicals’ Anti-Inflammatory and Anti-Cancer Effects

Although numerous studies demonstrate the beneficial effects of bitter phytochemicals on inflammation and cancer, and there is increasing evidence of TAS2Rs expressed across human cells, the direct connection between bitter phytochemicals and TAS2Rs in anti-inflammatory or anti-cancer actions remains relatively unexplored.

Evidence supporting the anti-inflammatory action of bitter phytochemicals through TAS2R involvement is limited. For instance, specific agonists activating TAS2Rs on mast cells have shown inhibitory effects on histamine and prostaglandin D2 (PGD2) release [298]. Studies by Zhang et al. revealed the inhibitory effect of bitter compounds, acting through TAS2R14, on IgE-induced mast cell degranulation [299]. Additionally, resveratrol was found to inhibit IL-6 release, a well-known pro-inflammatory cytokine, in human gingival fibroblasts through *TAS2R50* involvement [32]. Another example is the inhibition of pro-inflammatory cytokine release in human lung macrophages through bitter compounds targeting the TAS2R pathway [31].

Bitter compounds’ anti-cancer effects through TAS2Rs have been highlighted in various studies. Seo et al. showed how TAS2R8 and TAS2R10 suppressed cancer stemness by impeding self-renewal capacity and tumorigenicity in neuroblastoma cells while stimulating differentiation [97]. Similarly, noscapine exhibited anti-cancer actions through TAS2R14 in epithelial ovarian and prostate tumor cells, impacting cell survival [76]. Furthermore, denatonium, acting via the TAS2R pathway, affected leukemia cell survival, proliferation, migration, clonogenic potential, and the cell cycle [300]. Various bitter compounds induce cancer cell apoptosis in head and neck squamous cell tumors by targeting different TAS2Rs [35].

The specific roles of bitter phytochemicals in modulating the TAS2R pathway and their influence on the progression from chronic skin inflammation to skin cancer remain to be investigated in cell culture and in vivo studies.

## 10. Conclusions and Future Perspectives

TAS2Rs are expressed in all the layers of human skin in a personalized manner. Differential skin expression of TAS2Rs depends on many inter-individual or intra-individual factors, including age, sex, body map, and sun exposure. Some TAS2Rs have confirmed functionality within the skin and its cell lines. Experimental studies have shown promising effects of bitter phytochemicals in skin aging, wound healing, inflammatory skin conditions, and skin cancers, both in vitro and in animal models.

Exploring the therapeutic potential of bitter phytochemicals in inflammatory skin conditions and their influence on the risk of skin cancer development in patients with skin inflammation may be an interesting research area. Another avenue of investigation could assess combinations of two or more bitter phytocompounds targeting the same TAS2R, aiming to identify potential synergistic effects in animal models of skin conditions.

The drawbacks associated with bitter phytochemicals remain relatively underexplored and necessitate comprehensive research.

Importantly, the potential of bitter natural compounds for treating skin ailments is extensive. Therefore, investigations into the direct action of bitter phytochemicals on TAS2Rs within the skin or skin cell lines are eagerly anticipated. Targeting TAS2Rs could introduce innovative therapeutic approaches that prove beneficial for patients with diverse skin diseases.

## Figures and Tables

**Table 1:** Expression profiles of bitter taste receptors in human skin.

*TAS2R* Type	*TAS2Rs* Expression in the Skin	
	Intact and Healthy Human Skin Samples	Cultured or Native Human Skin Cell Types
*TAS2R1*	variablemRNA and protein expression throughout the epidermis (weak in basal cells, increasing from the stratum spinosum to the stratum corneum, cytoplasmic distribution) (+) [66]; mRNA (-) [64]; face- mRNA (-) [65];	mRNA and protein expression in HaCaT keratinocytes (+) [66]; mRNA and protein expression in human primary keratinocytes (+) [66]
*TAS2R3*	variable transcript expression based on sex (+) [64]; face- transcript (+) [65]; higher transcript expression in the female suprapubic area [64]	-
*TAS2R4*	variable transcript expression based on sex (+) [64]; face- transcript (+) [65]; higher transcript expression in the female suprapubic area [64]	protein expression in scalp hair follicles (outer root sheath keratinocytes) (+) [104]
*TAS2R5*	variable transcript expression based on age (+) [64]; face- transcript (+) [65]	-
*TAS2R7*	Face-transcript (+), in some specimens [65]; transcript (-) [64]	-
*TAS2R8*	variable transcript expression across samples based on sex (+) [64]; transcript (+)-face, in some specimens [65]; higher transcript expression in female suprapubic area [64]	_
*TAS2R9*	variable transcript expression based on sex (+) [64], face in some specimens- transcript (+) [65]	-
*TAS2R10*	universal transcript expression among individuals (+) [64]; face- transcript (+) [65]	transcript and protein expression in HaCaT cells (+) [105]
*TAS2R13*	variable transcript expression among individuals (+) [64]; face- transcript (+) [65]	-
*TAS2R14*	variable transcript expression based on location on body map (+) [64]; face- transcript (+) [65]; transcript and protein expression in abdominal skin (+) [42]	transcript expression in primary basal keratinocytes (+) [42]; transcript and protein expression in undifferentiated and differentiated N/TERT1 keratinocytes (+) [42]
*TAS2R16*	variable transcript expression among individuals (+) [64]; face- transcript (-) [65]	transcript and protein expression in HaCaT cells (+) [105];
*TAS2R19*	universal transcript expression among individuals (+) [64]; face- transcript (+) [65]	-
*TAS2R20*	variable transcript expression among individuals (+) [64]; face- transcript (+) in some specimens [65]	-
*TAS2R30*	universal transcript expression among individuals, variable abundance based on location on body map (+) [64]; face- transcript (+) [65]	-
*TAS2R31*	universal transcript expression among individuals (+) [64]; face- transcript (+), the highest transcript expression among the 25 TAS2Rs [65]	
*TAS2R38*	variable transcript expression among individuals (+) [64]; variable mRNA and protein expression throughout epidermis (+) (weak in basal cells, increasing from the stratum spinosum to the stratum corneum, cytoplasmic distribution) [66]; face- transcript (+) in some specimens [65]; transcript and protein expression in subcutaneous tissue samples (+) [106]; protein expression in mucous membranes (palate, tongue) [73]; transcript expression detected, protein expression barely detected in healthy skin (+) [107]	mRNA and protein expression (+) in HaCaT keratinocytes [66,73]; mRNA and protein expression (+) in human primary keratinocytes [66]; protein expression in fibroblasts (+) [73]; transcript and protein expression in isolated subcutaneous adipocytes (higher expression in obese subjects than lean ones) (+) [106]; transcript and protein expression in skin infiltrating T lymphocytes (lesional skin samples from patients with atopic dermatitis) (+) [107];
*TAS2R39*	variable transcript expression among individuals (+) [64]; face- transcript (+) in some specimens [65]	
*TAS2R40*	variable transcript expression among individuals (+) [64]; face- transcript (+) [65]	-
*TAS2R41*	variable transcript expression among individuals (+) [64]; face- transcript (+) [65]	-
*TAS2R42*	universal transcript expression among individuals, variable transcript abundance based on location on body map (+) [64]; face- transcript (+) [65]	-
*TAS2R43*	variable transcript expression among individuals (+) [64]; face- transcript (+) [65]	
*TAS2R45*	variable transcript expression among individuals (+) [64]; face- transcript (+) [65]	-
*TAS2R46*	variable transcript expression among individuals (+) [64]; face- transcript (+) [65]	-
*TAS2R50*	variable transcript expression among individuals (+) [64]; face- transcript (+) [65]	-
*TAS2R60*	universal transcript expression among individuals; variable transcript abundance based on sex and location on body map (+) [64]; face- transcript (+) [65]	-

Legend: *TAS2Rs*-bitter taste receptor gene; (+): *expression* present; (-): expression absent/not detected.

**Table 2:** Functionality of bitter taste receptors in the skin and oral cavity.

TAS2R Type	TAS2R Potential Functions in Skin and Oral Cavity
TAS2R1	Influence on the skin barrier proteins, lipid synthesis, and functionality [66]
TAS2R3	*TAS2R3* genetic variants associated with abundance in oral bacteria *Bergeyella* sp. HMT 907 [110]
TAS2R4	Inhibition of hair growth [104]; related to an increased survival rate in head and oral squamous carcinoma [35]
TAS2R5	?
TAS2R7	Oral detection of bitter salts (e.g., magnesium sulfate) [112]
TAS2R8	?
TAS2R9	*TAS2R9* genes are associated with the function of acetaldehyde dehydrogenase 1 in keratinocytes as well as atopic dermatitis pathogenesis [113]
TAS2R10	Inhibition of senescence of keratinocytes, activation of wound healing [105]
TAS2R13	?
TAS2R14	Chemosensory receptor, signaling the presence of noxious compounds in the skin [42]; longer survival in distantly metastatic skin melanoma cases [87]; increased oral innate immunity by detecting quorum sensing molecules released by the cariogenic bacteria (*Streptococcus mutans* and *Staphylococcus aureus*) [114,115]
TAS2R16	Inhibition of senescence of keratinocytes, activation of wound healing [105]
TAS2R19	?
TAS2R20	?
TAS2R30	?
TAS2R31	?
TAS2R38	Influence on the skin barrier proteins and lipids synthesis and functionality [66]; skin immunomodulatory activity [107]; adipocyte differentiation [106]; *TAS2R38* mutations among the most frequent non-silent mutations in skin melanoma [87]; *TAS2R38* genetic variant associated with abundance of oral *Candida albicans* [110].
TAS2R39	?
TAS2R40	?
TAS2R41	*TAS2R41* mutations among the most frequent non-silent mutations in skin melanoma [87]; a genetic variant associated with abundance in the oral fungus *Candida dubliniensis* [110]
TAS2R42	?
TAS2R43	?
TAS2R45	?
TAS2R46	Detection of noxious compounds (e.g., strychnine) [116]
TAS2R50	Inhibition of pro-inflammatory cytokines (e.g., IL6) in human gingival fibroblasts [32]
TAS2R60	*TAS2R60* mutations among the most frequent non-silent mutations in skin melanoma [87]

Legend. ?—not yet studied.

**Table 3:** Potential indications and disadvantages of bitter phytochemicals.

Phytocompound	TAS2R	Potential Indications (Evidence Derived from In Vivo and In Vitro Studies)	Disadvantages (Evidence Derived from In Vivo and In Vitro Studies)
Apigenin	TAS2R14 [132,142] TAS2R39 [132]	Psoriasis [154] Atopic dermatitis [155] Melanoma [156,157,158,159,160,161,162]	Negative impact on the reproductive system [273,274] Low water and non-polar solubility [275] Poor oral bioavailability [276]
Amarogentin	TAS2R1 [43],TAS2R4 [43], TAS2R39 [43],TAS2R43 [43], TAS2R46 [43],TAS2R47 [43], TAS2R50 [43]	Atopic dermatitis [167]	Liver and renal toxicity [277]
Amygdalin	TAS2R16 [43,170]	Psoriasis (amygdalin analogues) [174,175,176]	Toxicity due to hydrogen cyanide (amygdalin) [173]
Berberine	TAS2R38 [177] TAS2R46 [178]	Atopic dermatitis [179] Melanoma [181,182] Squamous cell carcinoma [180]	Gut microbiota dysbiosis [278] Diarrhea [278]
Chrysin	TAS2R14 [132] TAS2R39 [132]	Psoriasis [188] Atopic dermatitis [189] Melanoma [190,191,192,193,194,195,196,197]	Alteration in hematological parameters [279] Hepatic toxicity [279] Poor bioavailability [280]
Cucurbitacin B	TAS2R10 [43] TAS2R14 [43]	Psoriasis [199] Squamous cell carcinoma [200] Melanoma [201,202,203]	Low oral bioavailability [281] Non-selective toxicity [282]
Epigallocatechin gallate	TAS2R14 [132,204] TAS2R31 [204] TAS2R39 [132,204]	Psoriasis [206] Atopic dermatitis [207] Melanoma [208,209,210,213,214,215] Squamous cell carcinoma [211]	Hepatotoxicity [283,284,285] Dyslipidemia [285] Blocked anticancer effects of an anticancer drug (bortezomib) [286]
Genistein	TAS2R14 [132] TAS2R39 [132]	Psoriasis [227] Atopic dermatitis [228] Melanoma [218,219,220,221,222,223,224]	Negative impact on the reproductive system [287]
Kaempferol	TAS2R14 [132] TAS2R39 [132]	Psoriasis [230] Atopic dermatitis [231] Melanoma [232,233,234,235]	Genotoxicity in high doses [288]
Luteolin	TAS2R14 [132] TAS2R39 [132]	Atopic dermatitis [236] Psoriasis [237] Melanoma [238,239,240,241,242,243,244]	Negatively affects neuronal differentiation [289]
Naringenin	TAS2R14 [52,247]	Atopic dermatitis [248] Melanoma [249,251]	Negative impact on the reproductive system [290]
Resveratrol	TAS2R14 [132] TAS2R39 [132]	Psoriasis [253] Atopic dermatitis [254,255] Melanoma [256,257,258,260,261,262,263,264,265,266,267,270] Squamous cell carcinoma [259,271]	Poor bioavailability [254] Rapidly metabolized and not efficient in animal models of melanoma [291]

Legend: TAS2Rs-bitter taste receptors. Note: The activities/effects described in the third and fourth columns may not necessarily be mediated through TAS2R.

## Data Availability

Not applicable.
